# An Unusual Variant of a Calcifying Epithelial Odontogenic Tumor in the Maxilla: A Case Report

**DOI:** 10.7759/cureus.98686

**Published:** 2025-12-08

**Authors:** Subhasish Burman, Asish K Das, Sanjib Gain, Abhishek Khatua, Diptangshu Mallick, Srihari S, Saharsh Sarawgi

**Affiliations:** 1 Oral and Maxillofacial Surgery, Dr. R. Ahmed Dental College and Hospital, Kolkata, IND; 2 Oral and Maxillofacial Pathology, Dr. R. Ahmed Dental College and Hospital, Kolkata, IND

**Keywords:** calcifying epithelial odontogenic tumor, clear cell changes, leisegang's rings, macrocystic type, pindborg tumor

## Abstract

Calcifying epithelial odontogenic tumor (CEOT), also known as Pindborg tumor, is a rare benign odontogenic neoplasm that exhibits locally aggressive behavior. Histologically, it is characterized by sheets and nests of polyhedral epithelial cells with eosinophilic or, less frequently, clear cytoplasm. Other hallmark features include extracellular amyloid-like deposits, concentric calcifications known as Liesegang rings, and nuclear pleomorphism without significant mitotic activity. CEOTs are extremely uncommon in the maxilla, but when present in this location, they tend to exhibit more aggressive biological behavior and a higher recurrence rate compared to those in the mandible. We report a rare case of the macrocystic variant of CEOT in a 26-year-old female patient. Histopathological examination of the incisional biopsy revealed epithelial cell proliferation with eosinophilic, clear, and vacuolated cytoplasm interspersed with foci of amyloid-like material. Following diagnosis, complete enucleation of the tumor was performed under local anesthesia. The relevance of the clear cell variant of CEOT lies in its rarity, potential for more aggressive behavior, and diagnostic challenges. While the presence of clear cells in an odontogenic tumor is unusual, it can indicate a higher chance of recurrence and necessitates careful histopathological diagnosis to avoid confusion with other clear cell neoplasms, guiding more aggressive treatment and follow-up.

## Introduction

First identified by Pindborg [1] in 1955, the calcifying epithelial odontogenic tumor (CEOT), commonly known as the Pindborg tumor [2], is an uncommon benign odontogenic tumor of the jaws. In their analysis of 181 Pindborg tumor cases, Philipsen and Reichart [3] found that 15 of them had lesions that contained clear cells. Two entities have been identified based on topography: the intra-osseous (central) and the extra-osseous (peripheral) Clear cell variety [4-14]. A rare benign odontogenic tumor with the potential for local invasiveness, CEOT has been well documented in the literature. CEOT is generally characterised as a benign, locally aggressive tumor that grows slowly. According to Hicks et al., the clear cell variation can exhibit more aggressive behavior and can have a greater recurrence rate (22%) [13].

In adults, clear cells can be detected as clear-cell rests of the dental lamina within the connective tissue wall of lateral periodontal and gingival cysts, or they might be present as cellular components of the epithelial lining of these cysts. CEOT has been categorised by some writers as a distinct clinical entity. Rarely, some epithelial odontogenic tumors [15] may have transparent cells.

The first instance of CEOT primarily consisting of transparent cells was reported by Abrams and Howell in 1967 [4]. Two of those 23 CEOT patients were deemed diagnostically challenging by Krolls and Pindborg a few years later because of their high clear cell concentration [3,14,16,17-20]. Since then, the clear cell component's predominance in CEOTs has mostly been documented through individual instances, and its significance for prognosis is still up for debate.

A CEOT is classified based on its location and histological features. Its main classification is intraosseous (central), which is the most common type, or extraosseous (peripheral). Recent classifications also include histological variants such as clear cell, cystic/microcystic, and noncalcifying.

## Case presentation

**Figure 1:**
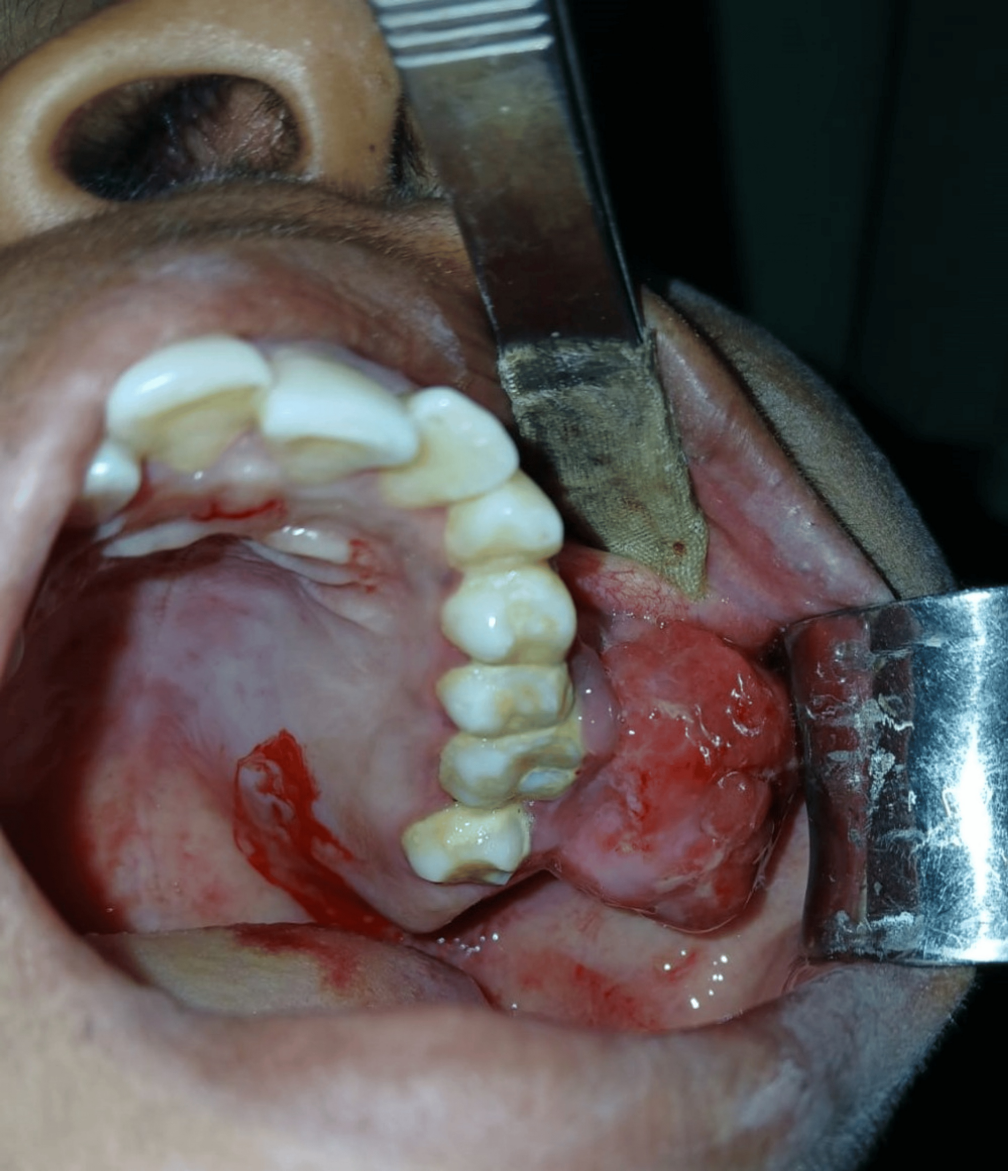
Intraoral photograph showing solitary swelling in the left posterior maxillary region.

A young female patient aged 26 years reported to our institute with a complaint of a painless enlargement of her upper left posterior jaw for three months, which was gradually increasing in size. On the left posterior maxillary area, the growth was approximately 3.5 x 2.5 cm in size, and the involved adjacent teeth were supra-eruptive, with normal color of adjacent mucosa (Figure 1). On palpation, it was non-tender and hard in consistency. In addition, teeth 27 and 28 were also impacted, and local periodontitis was noted in teeth 24, 25, and 26.

**Figure 2:**
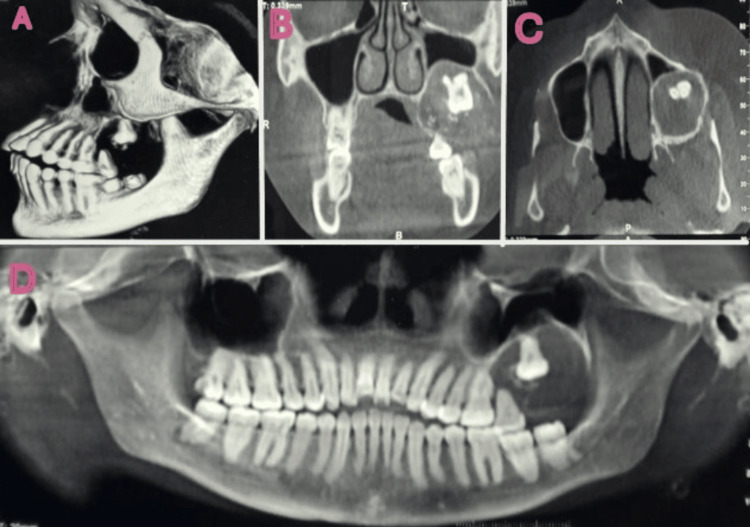
Snow-driven mixed radiolucent and radiodense lesion in the left maxilla as seen in the orthopantomogram (OPG) A: 3D image; B: coronally sectioned image; C: an axially sectioned image; D: OPG image; the OPG revealed a well-defined, 4 × 3.5 cm mixed radiopaque-radiolucent lesion in the posterior maxilla with regional impacted teeth.

A single, hard intraosseous enlargement was palpable during the intraoral examination with a little buccal and palatal cortical plate expansion. The orthopantomogram (OPG) revealed a well-defined, 4 × 3.5 cm mixed radiopaque-radiolucent lesion in the posterior maxilla with regional impacted teeth (Figure 2).

Cone beam computed tomography (CBCT) revealed a radiolucent-radiopaque mass in the left maxilla invading the cortex. The lesion extended to the posterior maxilla and obliterated the left nasal cavity. The radiographic differential diagnosis of the lesion included lateral periodontal cyst, glandular odontogenic cyst, early-stage ossifying fibroma, central odontogenic fibroma, and extrafollicular adenomatoid odontogenic tumor.

**Figure 3:**
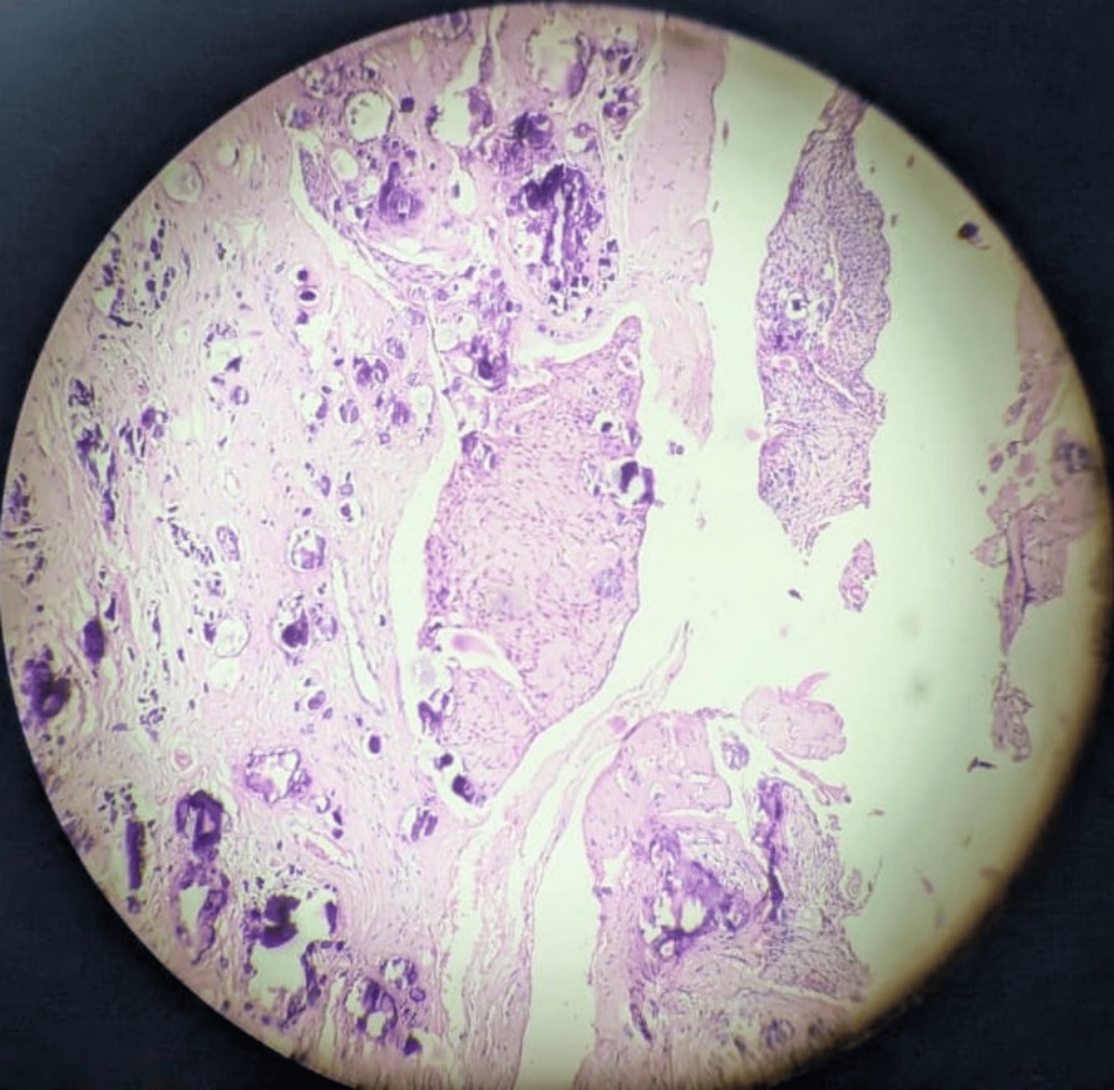
Histopathological features confirming the diagnosis of calcifying epithelial odontogenic tumor Hematoxylin and eosin-stained sections showed the presence of a fibrocollagenous connective tissue capsule, showing the presence of multiple tumor islands of varying sizes and discrete or multiple calcifications in the form of Liesegang's rings. Tumor islands were composed of polygonal cells with prominent intracellular bridges, along with some clear cell (clear vacuolated cytoplasm) changes. A small cystic lining was appreciated in a few sections. Another hematoxylin and eosin-stained section showed multiple bits of soft tissue showing large sheets of tumor cells as described above, along with amyloid changes and a few calcified areas. The amyloid material was positive for Congo red staining, which exhibited apple-green birefringence under polarized light analysis

An incisional biopsy was taken from the suspected site. The specimen was sent for histopathological examination to the Department of Oral Pathology. Hematoxylin and eosin-stained sections showed the presence of a fibrocollagenous connective tissue capsule, showing the presence of multiple tumor islands of varying sizes and discrete or multiple calcifications in the form of Liesegang's rings. Tumor islands were composed of polygonal cells with prominent intracellular bridges, along with some clear cell (clear vacuolated cytoplasm) changes. A small cystic lining was appreciated in a few sections. Another hematoxylin and eosin-stained section showed multiple bits of soft tissue showing large sheets of tumor cells as described above, along with amyloid changes and a few calcified areas. The amyloid material was positive for Congo red staining, which exhibited apple-green birefringence under polarized light analysis (Figure 3). The final diagnosis was of the clear cell variant of CEOT.

Given the patient's age, the benign nature of CEOT, and aesthetic concerns, curettage of the lesion was carried out along with the removal of the related impacted tooth under local anesthesia. After the curettage of the lesion, the histopathological examination was done, and the diagnosis was made as the macrocystic variant of CEOT.

**Figure 4:**
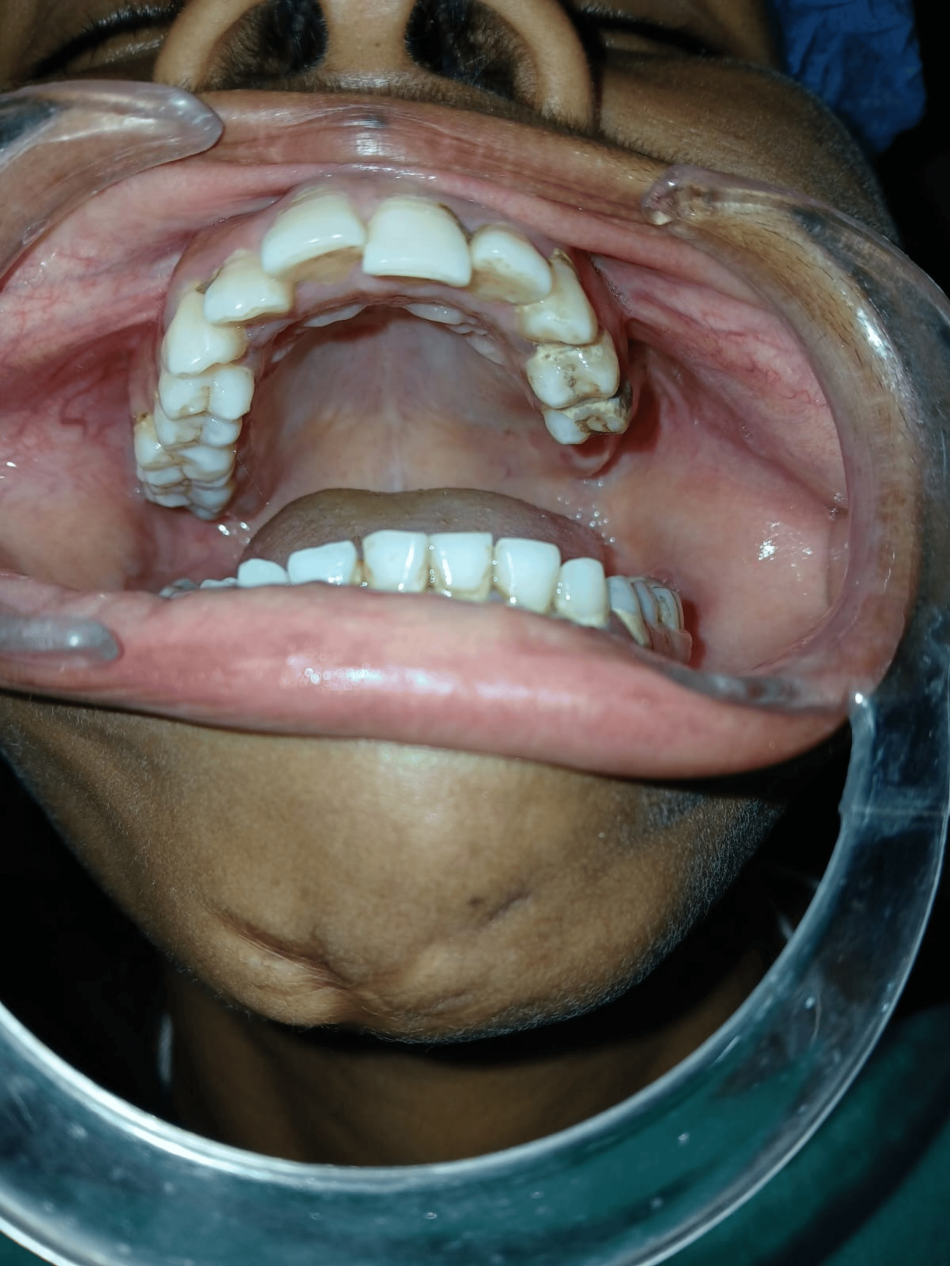
No evidence of recurrence clinically

**Figure 5:**
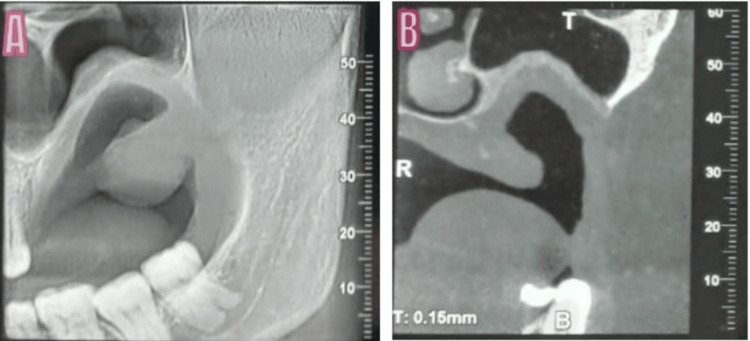
No evidence of recurrence in the cone-beam computed tomography (CBCT) reports in the six-month follow-up

No recurrence was found on the six-month follow-up of the patient (Figures 4, 5)

## Discussion

Although some authors have suggested that CEOT composed primarily of clear cells may follow a more aggressive clinical course with an increased rate of recurrence, the proper clinical relevance regarding its biological behavior remains debatable. Fewer than 40 cases of the clear cell variant of CEOT have been documented in the literature to date. The presence of numerous clear cells in CEOT has been widely recognized in the literature since the first two cases of this variant were reported by Abrams and Howell in 1967 [4]. Anavi et al. [18] conducted a thorough assessment of the literature on the clinical and radiologic characteristics of CEOT, documenting 19 cases to date, including 12 central and seven peripheral lesions. Of the 12 central lesions, nine have been reported to involve the mandible and three to involve the maxilla (75:25), with an equal distribution between males and females and an age range of 14-68 years [18].

The most common clinical manifestation of CEOT is cortical enlargement brought on by painless jaw edema. Unilocular or multilocular lesions with mixed radiolucent and radiopaque areas of varying densities are radiological characteristics of CEOT. Descriptions of a "driven snow" or "wind-driven snow" appearance have been reported. It is usually associated with an impacted tooth. The radiological and clinical features of the present case match those of previously published CEOT cases. In this case study, the lesion demonstrated a typical driven snow appearance, whereas the three maxillary lesions previously described were radiolucent on OPG [10,12,14].

Histological sections of CEOT revealed fibrocollagenous connective tissue. Numerous tumor islands of varying sizes with discrete or multiple calcifications were present within the capsule. These tumor islands were composed of polygonal cells with persistent intercellular bridges and some clear cell changes (transparent vacuolated cytoplasm). A small cystic lining was also observed in some sections [14]. Other sections of the soft tissue showed large sheets of tumor cells with amyloid changes and a few calcified areas. Under polarized light, the amyloid material exhibited apple-green birefringence, indicating positive Congo red staining.

Microscopic differential diagnoses of CEOT predominantly composed of clear cells include central mucoepidermoid carcinoma and metastatic lesions from the kidney, thyroid, and lung, as well as other odontogenic tumors such as ameloblastoma and clear cell odontogenic carcinoma [14,18,19]. In this case, the final diagnosis of clear cell predominant CEOT was established based on the definitive presence of amyloid material and calcified formations, the absence of microscopic ameloblastomatous differentiation, and the lack of clinical or radiographic evidence of malignant disease [14].

Compared to conventional CEOT, the clear cell variant is more aggressive and has a higher recurrence rate. Treatment options range from conservative methods such as enucleation or curettage to more extensive surgical resections. Among the 19 documented cases, surgical treatment included curettage in one case (5%), enucleation in four cases (21%), excision in seven cases (37%), and total or partial resection in seven cases (37%). Two cases of recurrence (17%) have been reported [18].

According to studies by Hicks et al. [13], the presence of clear cells may indicate a more aggressive tumor and justify more radical treatment. In our case, there is no strong evidence to support aggressiveness, as the tumor did not recur following enucleation.

There is proof that the clear cell variation is a unique entity with a higher likelihood of recurrence and more aggressive biological behavior. Therefore, the inclusion of clear cells in the histological diagnosis is crucial. The presence of a transparent cell helps the surgeon remove the lesion with greater certainty. Because maxillary lesions grow more quickly and are located close to essential tissues, they should be treated more aggressively.

## Conclusions

The present case highlights a rare presentation of the macrocystic variant of CEOT involving the posterior maxilla, an uncommon site for this already rare odontogenic neoplasm. While CEOT more frequently involves the mandible and typically demonstrates a solid growth pattern, this case exhibited a prominent macrocystic architecture, contributing to a unique clinical and radiographic appearance. The lesion presented as a painless, slowly enlarging swelling, with imaging revealing a unilocular radiolucency exhibiting scattered radiopacities, consistent with the characteristic “driven snow” appearance seen in some CEOTs. Surgical enucleation was chosen as the treatment modality based on the well-defined margins, lack of cortical perforation, and absence of invasion into surrounding vital structures.

This case emphasizes the importance of considering CEOT in the differential diagnosis of radiolucent-radiopaque lesions in the posterior maxilla, particularly when a cystic pattern is observed. It also reinforces the histological diversity of CEOT, including rare variants like the macrocystic type, which can pose diagnostic challenges. Although the macrocystic variant is rare, awareness of its existence is crucial for accurate diagnosis and appropriate management.

## References

[1] Ranlov P, Pindborg JJ (1966). The amyloid nature of the homogeneous substance in the calcifying epithelial odontogenic tumour. Acta Pathol Microbiol Scand.

[2] Shafer WG, Hine MK, Levy BM (1963). A Textbook of Oral Pathology, 2nd Edition. Philadelphia: W.B Saunders.

[3] Philipsen HP, Reichart PA (2000). Calcifying epithelial odontogenic tumour: biological profile based on 181 cases from the literature. Oral Oncol.

[4] Abrams AM, Howell FV (1967). Calcifying epithelial odontogenic tumors: report of four cases. J Am Dent Assoc.

[5] Anderson HC, Kim B, Minkowitz S (1969). Calcifying epithelial odontogenic tumor of Pindborg. An electron microscopic study. Cancer.

[6] Greer RO Jr, Richardson JF (1976). Clear-cell calcifying odontogenic tumor viewed relative to the Pindborg tumor. Oral Surg Oral Med Oral Pathol.

[7] Wallace J, MacDonald GD (1974). Calcifying epithelial odontogenic tumour ("Pindborg tumour"): a case report. Br J Plast Surg.

[8] Oikarinen VJ, Calonius PE, Meretoja J (1976). Calcifying epithelial odontogenic tumor (Pindborg tumor) case report. Int J Oral Surg.

[9] Yamaguchi A, Kokubu JM, Takagi M, Ishikawa G (1980). Calcifying epithelial odontogenic tumor: histochemical and electron microscopic observations on a case. Bull Tokyo Med Dent Univ.

[10] Asano M, Takahashi T, Kusama K (1990). A variant of calcifying epithelial odontogenic tumor with Langerhans cells. J Oral Pathol Med.

[11] Schmidt-Westhausen A, Philipsen HP, Reichart PA (1992). Clear cell calcifying epithelial odontogenic tumor. A case report. Int J Oral Maxillofac Surg.

[12] Takata T, Ogawa I, Miyauchi M, Ijuhin N, Nikai H, Fujita M (1993). Non-calcifying Pindborg tumor with Langerhans cells. J Oral Pathol Med.

[13] Hicks MJ, Flaitz CM, Wong ME, McDaniel RK, Cagle PT (1994). Clear cell variant of calcifying epithelial odontogenic tumor: case report and review of the literature. Head Neck.

[14] Kumamoto H, Sato I, Tateno H, Yokoyama J, Takahashi T, Ooya K (1999). Clear cell variant of calcifying epithelial odontogenic tumor (CEOT) in the maxilla: report of a case with immunohistochemical and ultrastructural investigations. J Oral Pathol Med.

[15] Rasmusson LG, Magnusson BC, Borrman H (1991). The lateral periodontal cyst. A histopathological and radiographic study of 32 cases. Br J Oral Maxillofac Surg.

[16] de Oliveira MG, Chaves AC, Visioli F (2009). Peripheral clear cell variant of calcifying epithelial odontogenic tumor affecting 2 sites: report of a case. Oral Surg Oral Med Oral Pathol Oral Radiol Endod.

[17] Rangel AL, da Silva AA, Ito FA, Lopes MA, de Almeida OP, Vargas PA (2009). Clear cell variant of calcifying epithelial odontogenic tumor: is it locally aggressive?. J Oral Maxillofac Surg.

[18] Anavi Y, Kaplan I, Citir M, Calderon S (2003). Clear-cell variant of calcifying epithelial odontogenic tumor: clinical and radiographic characteristics. Oral Surg Oral Med Oral Pathol Oral Radiol Endod.

[19] Bhambal AM, Trivedi A, Deshmukh P, Shivakumar GC (2022). Calcifying epithelial odontogenic tumour of the maxilla - a rare case report. J Oral Maxillofac Pathol.

[20] Pourdanesh F, Armanfar M, Mashhadiabbas F, Gholami S, Mohammadalizadeh Chafjiri M, Khorsand A (2024). Huge calcifying epithelial odontogenic tumor of the mandible and management with a teeth preserving surgical approach: a case report. J Med Case Rep.

